# Decoding HIV-associated neurocognitive disorders: a new perspective from multimodal connectomics

**DOI:** 10.3389/fneur.2025.1467175

**Published:** 2025-01-29

**Authors:** Zhongkai Zhou, Wei Wang, Hui Li, Ying Shi, Lingling Zhao, Yibo Lu, Xingchen Wei, Hongjun Li

**Affiliations:** ^1^Department of Radiology, Beijing Youan Hospital, Capital Medical University, Beijing, China; ^2^Department of Neurology, Xuanwu Hospital, Capital Medical University, Beijing, China; ^3^Department of Magnetic Resonance, The First Affiliated Hospital of Harbin Medical University, Harbin, China; ^4^Department of Radiology, The Sixth People's Hospital of Zhengzhou, Zhengzhou, China; ^5^Department of Radiology, The Fourth People's Hospital of Nanning, Nanning, China; ^6^Department of Joint Surgery, Shandong Provincial Hospital, Jinan, China

**Keywords:** connectomics, HIV-associated neurocognitive disorders, structural connectivity, functional connectivity, default mode network

## Abstract

Currently, HIV-associated neurocognitive disorders (HAND) remains one of the major challenges faced by people living with HIV (PLWH). HAND involves the vulnerability of neural circuits caused by synaptic degeneration and abnormal synaptic pruning. In recent years, connectomics has been gradually applied to HAND research as a cutting-edge method for describing the structural and functional connectivity patterns of the brain, to further elucidate the specific mechanisms underlying these neural circuit vulnerabilities. Using multimodal neuroimaging techniques such as diffusion tensor imaging (DTI), structural magnetic resonance imaging (sMRI), and resting-state functional magnetic resonance imaging (rs-fMRI), researchers can detail the connectome network changes in the brains of PLWH. These technologies offer potential biomarkers for the early diagnosis, prognosis, and treatment monitoring of HAND, while also providing new avenues for personalized prediction of cognitive status. Here, we start with the pathogenesis and risk factors of HAND, providing a comprehensive review of the basic concepts of unimodal and multimodal macro connectomics and related graph theory methods, and we review the latest progress in HAND connectomics research. We emphasize the use of connectomics to identify specific disease patterns of HIV-associated neurodegeneration and discuss the potential research directions and challenges in understanding these diseases from a connectomics perspective.

## 1 Introduction

According to global statistics and the World Health Organization in 2023, approximately 39 million people are living with HIV, among whom nearly 76% are receiving antiretroviral therapy (ART) (1). ART has significantly extended the lifespan of people living with HIV (PLWH), transforming HIV from a fatal disease into a manageable chronic condition (2). However, with prolonged survival, PLWH face the challenge of chronic complications, among which HIV-associated neurocognitive disorders (HAND) is both common and particularly challenging. HAND can manifest as impairments in executive function, attention, and memory, significantly reducing patients’ quality of life and independence (3, 4).

According to the Frascati diagnostic criteria, HAND can be categorized into three types based on disease severity: asymptomatic neurocognitive impairment (ANI), mild neurocognitive disorder (MND), and HIV-associated dementia (HAD) (5). The latest meta-analysis shows that the global prevalence of HAND among PLWH is as high as 42.6%, with ANI being the most common subtype, accounting for 28.4% (6). Although the widespread use of ART has significantly reduced the incidence of HAD, the proportions of ANI and MND have not declined. This suggests that mild HAND remains an unresolved clinical challenge even under long-term viral suppression (7–9). Furthermore, ANI is considered an early stage of HAND, with potentially reversible pathological changes. However, the risk of progressing to symptomatic disorders is 2–6 times higher in individuals with ANI compared to typical HIV-infected individuals (10).

Currently, the diagnosis of HAND primarily relies on neuropsychological (NP) tests, which cover at least five cognitive domains: attention/working memory, executive function, learning and memory, language abilities, and visuospatial/perceptual-motor skills (5). Although NP tests exhibit high sensitivity and specificity, they are time-consuming, depend on professional evaluation, and are susceptible to practice effects, which limits their widespread clinical application. Therefore, identifying sensitive and practical objective biomarkers to optimize early diagnostic strategies for HAND has become a key focus. In recent years, non-invasive magnetic resonance imaging (MRI) techniques such as diffusion tensor imaging (DTI), structural magnetic resonance imaging (sMRI), and resting-state functional magnetic resonance imaging (rs-fMRI) have played crucial roles in HAND research. These techniques can identify microstructural and functional abnormalities before the onset of clinical symptoms, providing valuable insights into the pathological mechanisms of HAND (11).

With the continuous advancement of MRI neuroimaging and its derived technologies, it has become possible to map brain structural and functional connectivity at a macroscopic scale, offering new opportunities to better understand neural network damage and connectivity pattern changes in HAND. Connectomics, as a cutting-edge approach to studying neural network integrity, provides critical insights into the pathological mechanisms of HAND by quantifying the structural and functional connectivity properties of brain networks (12). This approach can uncover the profound impacts of HIV infection on brain networks, including structural and functional reorganization, disruption of key pathways, and activation of compensatory networks. Connectomics has achieved significant progress in areas such as Alzheimer’s disease, psychiatric disorders, and autism spectrum disorder (13–15). Although the application of these techniques in HIV-related neuropathology remains in the exploratory stage, their potential to elucidate HAND’s neural mechanisms, identify potential diagnostic biomarkers, and optimize intervention strategies is gradually emerging.

This review begins with the pathogenic mechanisms and multifactorial risks of HAND, with a specific focus on connectomics research based on rs-fMRI, diffusion magnetic resonance imaging (dMRI), and sMRI. It systematically summarizes the key findings of recent studies on connectomics in HAND and explores its potential applications in early diagnosis, monitoring, and intervention. The article also analyzes the limitations of current technologies and proposes future directions, aiming to provide new insights and guidance for HAND-related research and clinical practice.

## 2 Methodology overview

The brain is regarded as a complex network composed of nodes and the edges connecting these nodes. To date, key topological properties such as small-world characteristics, modular structures, and hub nodes have been extensively validated across species and at multiple scales in both structural and functional networks (16, 17). These studies are fundamentally based on the methods of constructing and analyzing complex networks. Specifically, at the macroscopic scale, network nodes are first defined based on various prior atlases. Functional network connectivity (FCN) is then constructed by calculating the temporal correlation of rs-fMRI signals between nodes, while structural network connectivity (SCN) is derived from white matter fiber tracts traced using dMRI or from morphological parameter covariance at the group level, or morphological similarity at the individual level, based on sMRI data (18–20). Although FCN and SCN are constructed through different approaches, they exhibit high reproducibility and reliability in characterizing the key topological properties of brain networks. This further underscores the value of both functional and structural networks in elucidating the brain’s segregated and integrated features. Connectomics studies have elucidated various complex topological properties of healthy brain networks. For instance, high clustering coefficients and modular structures reflect the segregative properties of brain networks, whereas low characteristic path lengths, a prominent “rich-club” structure (i.e., dense interconnections among highly connected regions), and high nodal degree centrality indicate the integrative capacity of brain networks (21, 22).

In recent years, connectomics research has progressively adopted more refined and dynamic perspectives, offering new directions for unraveling brain functional mechanisms and improving disease diagnosis. In terms of data acquisition, high-angular-resolution diffusion imaging (HARDI) addresses the limitations of DTI in resolving fiber crossings, convergences, and twisted regions by acquiring more diffusion directions with higher angular resolution (23). In terms of data processing, mutual connectivity analysis (MCA) quantifies the nonlinear dependencies between time series, uncovering complex relationships that traditional Pearson correlation matrices cannot capture (24, 25). Granger causality analysis has emerged as a crucial tool for investigating directed connections in complex networks and time-series data (26). Moreover, unlike the assumption of stable static functional connectivity, dynamic functional connectivity (dFC) employs techniques such as sliding time windows to capture the dynamic fluctuations and transient characteristics of brain functional networks, revealing dynamic patterns associated with behavior or pathological states (27, 28). Advances in data acquisition and processing techniques not only provide robust support for analyzing the segregative and integrative properties of brain networks but also offer valuable insights into their pathological mechanisms and diagnostic potential.

It is widely accepted that brain function is constrained by its underlying anatomical structure and that structural and functional networks share common topological features, such as modular organization and hub nodes (29, 30). However, functional and structural networks also exhibit differences across cortical hierarchies, frequencies, and temporal scales (31–34). Nevertheless, integrating multiple neuroimaging modalities (e.g., structural and functional data) to construct unified brain network maps and explore the interactive mechanisms between modalities is crucial for comprehensively uncovering the structural and functional properties of the brain, as well as their relationships with behavior and pathological states. For example, Sarwar et al. (35) utilized deep learning methods to predict individual functional connectivity from structural connectomes, achieving highly accurate personalized predictions and successfully explaining significant inter-individual differences in cognitive performance. This study further supports the close interrelationship between structural and functional networks and demonstrates that integrating structural and functional modalities in research facilitates a more comprehensive understanding of brain topological properties and internal working mechanisms from dual perspectives.

## 3 Pathogenic mechanisms and multifactorial effects of HAND

### 3.1 Direct and indirect effects of HIV

**Figure 1:**
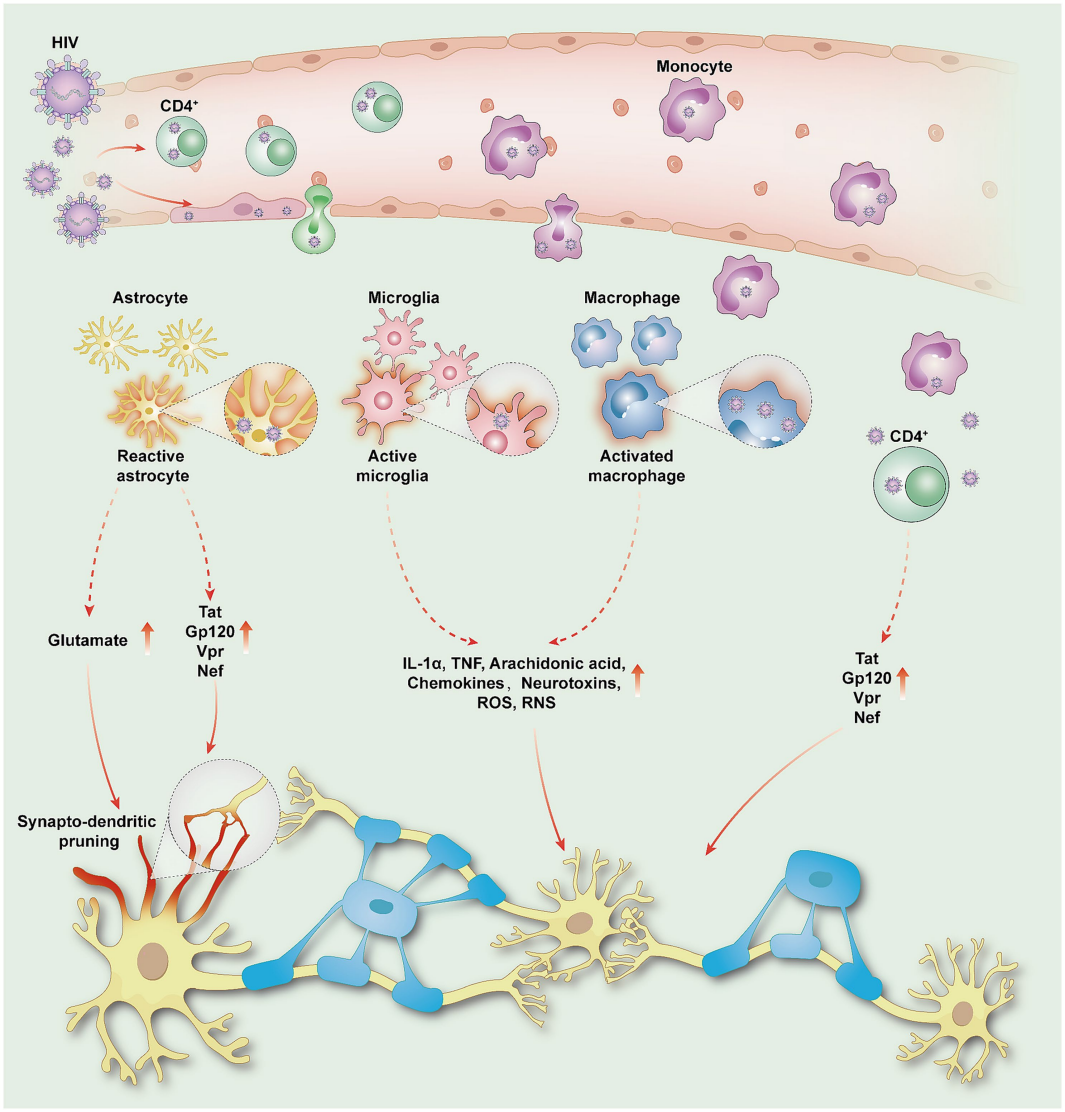
HIV penetrates the blood–brain barrier and enters the CNS, damaging neurons and inducing HAND. HIV enters the CNS either through transcytosis via epithelial cells or by passing through intercellular spaces via infected monocytes and CD4 lymphocytes. Once in the CNS, HIV releases viral proteins such as Tat, gp120, Vpr, and Nef, leading to oxidative stress reactions that directly damage neurons; Activated astrocytes promote elevated glutamate levels and release viral proteins, triggering synapto-dendritic pruning and weakening functional connectivity between neurons. Macrophages or microglia serve as significant reservoirs of the virus. Reactive astrocytes and activated microglia release inflammatory cytokines (IL-1α, TNF), arachidonic acid, chemokines, neurotoxins, ROS, and RNS, leading to inflammatory and neurotoxic cascades. HIV, human immunodeficiency virus; HAND, HIV-associated neurocognitive disorders; CNS, central nervous system; Tat, trans-activator of transcription; Gp120, glycoprotein 120; Vpr, viral protein R; Nef, negative regulatory factor; IL-1α, interleukin-1 alpha; TNF, tumor necrosis factor; ROS, reactive oxygen species; RNS, reactive nitrogen specie.

HIV enters the central nervous system (CNS) via the “Trojan horse” pathway, releasing neurotoxic proteins (such as Tat, gp120, Vpr, and Nef), which lead to oxidative stress and neuronal damage (36). Meanwhile, HIV infects macrophages or microglial cells, which indirectly exacerbate neuronal damage by releasing inflammatory cytokines and other neurotoxic substances (37). In addition, HIV activates astrocytes, leading to elevated glutamate levels, which result in reduced synaptic density and dendritic simplification, thereby weakening functional connections between neurons (38, 39). Such synaptic and neural circuit damage disrupts the integrity of brain networks, forming the core neuro-network basis of HAND (Figure 1).

### 3.2 Legacy effects and cerebrospinal fluid HIV ribonucleic acid escape

During the acute infection phase, HIV invades the CNS, triggering inflammation and initiating immune activation effects. This “legacy effect” persists even after ART effectively controls systemic viral replication (40). Due to drug resistance within the CNS and insufficient CNS penetration effectiveness (CPE) of ART, the phenomenon of “CSF HIV RNA escape” is not uncommon, making it difficult to suppress viral and inflammatory accumulation within the CNS (39, 41–44). The accumulated HIV further damages the blood–brain barrier (BBB), exacerbating chronic inflammation and persistent neuronal injury (45, 46). In summary, “legacy effect” and “CSF HIV RNA escape” result in prolonged exposure of neurons and brain networks to a chronic inflammatory and damaging environment, which may be a significant factor in the pathological progression of HAND.

### 3.3 External and synergistic factors

**Figure 2:**
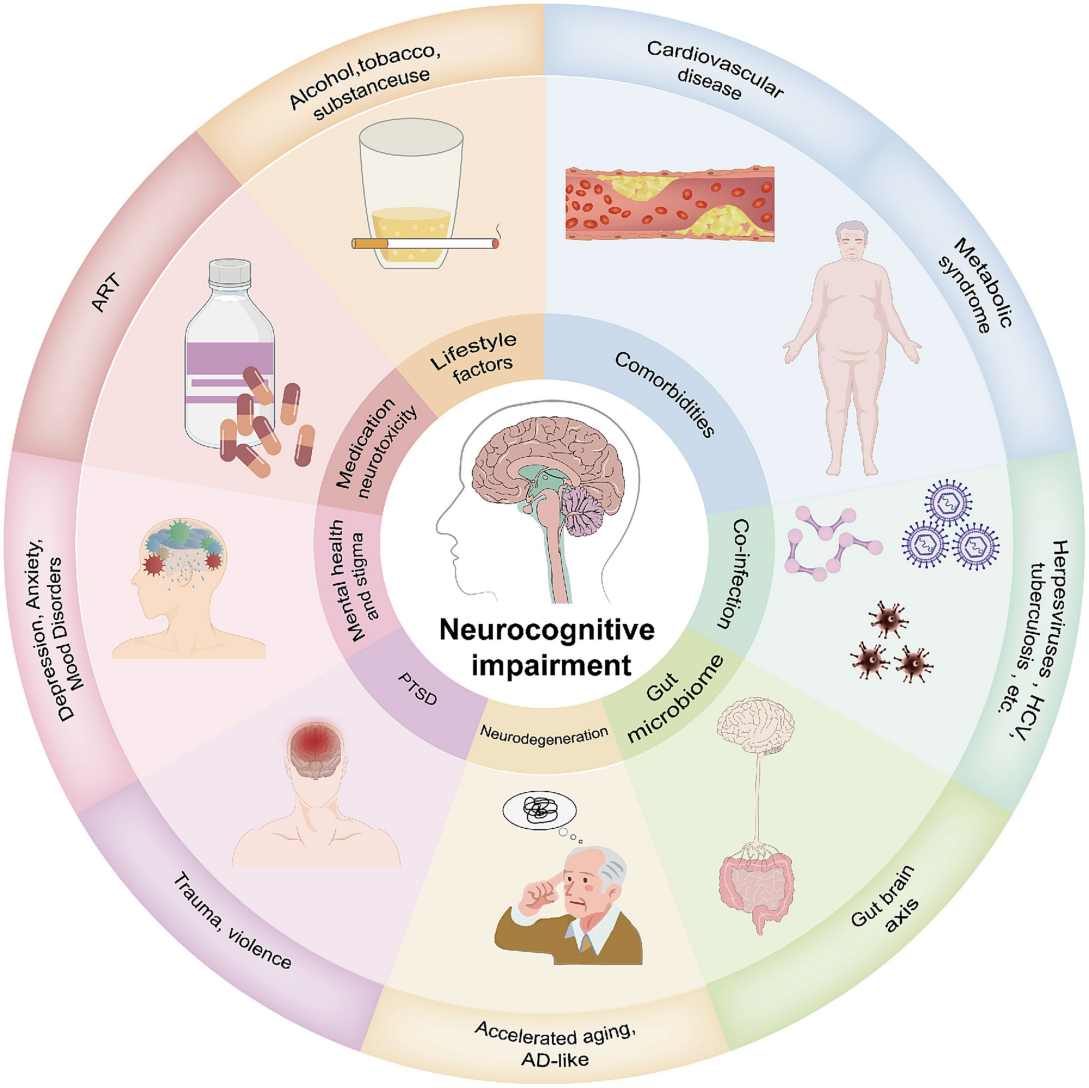
HAND involves multiple pathogenic factors. Several factors are considered active or ongoing insults to cognitive function, many of which are directly related to HIV infection and treatment. The summarized pathogenic mechanisms include: (1) Comorbidities such as cardiovascular diseases and metabolic syndrome; (2) Co-infections such as herpes virus, hepatitis C virus, and *Mycobacterium tuberculosis*; (3) The gut is an important reservoir for HIV and acts as a hub for its dissemination. Gut inflammation can lead to neuroinflammation; (4) Accelerated aging due to HIV, with HIV neurodegenerative mechanisms potentially similar to those of Alzheimer’s disease (AD); (5) PTSD due to childhood trauma and violence; (6) Mental health issues and stigma; (7) Antiretroviral drugs can directly or indirectly induce neurotoxicity; (8) Unhealthy lifestyles commonly found in PLWH, including smoking, substance abuse, and alcohol misuse.

The neurotoxicity of ART, aging, and HIV act synergistically to accelerate brain functional decline. Common comorbidities (such as hepatitis C, cardiovascular diseases, and mood disorders), as well as disruptions of the gut-brain axis, increase stress on the central nervous system. Unhealthy lifestyle habits (such as smoking, excessive alcohol consumption, and substance abuse) further exacerbate neural damage (47, 48). These factors collectively amplify the detrimental effects of HIV on brain networks, further complicating the pathogenesis of HAND (Figure 2).

### 3.4 Connectomics perspective

Based on the aforementioned multi-mechanistic pathological processes, HIV infection not only reduces synaptic density, simplifies dendritic structures, and damages white matter fibers but also destabilizes structural connectivity, alters the synchrony and efficiency of functional connectivity, and reshapes the topological properties of brain networks. These abnormalities manifest as changes in brain network integration and segregation, which are closely associated with cognitive decline in HAND. Connectomics offers a novel perspective for quantifying brain network properties, not only elucidating the neural mechanisms of HAND but also providing critical insights for the identification of diagnostic biomarkers and the development of intervention strategies. For instance, functional connectomics has uncovered the reorganization of core networks, structural connectomics has identified disruptions in critical pathways and the activation of compensatory networks, while multimodal connectomics has analyzed the complex pathological changes of HAND from a global network perspective. These research findings lay an important foundation for the precise diagnosis of HAND and the development of personalized treatment strategies.

## 4 Core findings of functional connectomics in HAND

### 4.1 The impact of HIV infection on core brain networks (default mode network, central executive network, salience network)

#### 4.1.1 Default mode network (DMN)

Studies have shown that the DMN in PLWH exhibits significant functional connectivity abnormalities, making it one of the most promising biomarkers for cognitive impairment. Abidin et al. (25, 49) using network-based statistics (NBS) and MCA, identified abnormalities in key DMN nodes [such as the posterior cingulate cortex (PCC), precuneus, and intraparietal sulcus (IPS)]. These abnormalities indicate a reorganization of the DMN’s topological structure, characterized by reduced modularity and small-world properties, which are associated with deficits in attention, memory, and executive function.

#### 4.1.2 Central executive network (CEN) and salience network (SN)

Flannery et al., through Dynamic Causal Modeling (DCM) analysis, found that the functional interaction between the SN and the DMN is significantly impaired in HAND patients, particularly in the PCC and prefrontal cortex regions. Further analysis indicated a significant reduction in modularity and small-worldnesss in HAND patients, suggesting a profound reorganization of the overall topological structure of brain networks. This dysfunction leads to disrupted allocation of cognitive resources, manifesting as an increase in executive function errors and a decrease in global network efficiency, reflecting abnormal connectivity characteristics of core functional networks in HAND patients (50). Moreover, Chaganti et al., using Independent Component Analysis (ICA), extracted the primary resting-state brain networks of HAND patients and healthy controls. The study revealed that the functional connectivity between the SN and CEN was significantly reduced, particularly in the prefrontal cortex and insular regions. This reduction was closely associated with impairments in executive function and attention in HAND patients (*r* = 0.34, *P* < 0.05) (51).

### 4.2 Factors influencing functional connectomes: aging, substance use, educational level, and immune status

#### 4.2.1 HIV infection and aging

Studies have shown that HIV infection and aging jointly weaken the connectivity of core functional networks, particularly the DMN, SN, and frontoparietal network (FPN). Egbert et al. (52) found that this effect primarily manifests as significantly weakened connectivity between the DMN and other networks, such as the dorsal attention network (DAN) and the sensorimotor network (SMN). Thomas et al. (53) emphasized that the decline in intra-network connectivity is closely associated with impairments in executive function and attention. Although neither study identified significant interaction effects, both suggested that HIV may accelerate brain network degeneration through mechanisms similar to aging, characterized by concurrent impairments in network integration and segregation, further highlighting the long-term impact of HIV on brain networks.

#### 4.2.2 HIV infection and substance use

The impact of substance use on the brain functional networks of individuals with HIV infection is complex. Cocaine use and HIV infection independently disrupt core networks, such as the DMN and CEN, resulting in reduced network integration and increased segregation. These alterations in network properties may further impair information processing efficiency and executive function (54). In contrast, cannabis use exhibits a regulatory effect on the brain networks of individuals with HIV infection. Studies have shown that cannabis enhances local connectivity while weakening long-range connections, partially mitigating network abnormalities in the context of HIV infection, and bringing functional connectivity closer to healthy levels (55). This suggests that the effects of different substances on brain networks are heterogeneous, with cocaine exacerbating network damage, while cannabis may have protective or compensatory effects in certain circumstances. However, the long-term effects of this regulatory mechanism on cognitive function require further investigation.

#### 4.2.3 The modulating role of educational attainment

Higher educational attainment can alleviate the negative effects of HIV infection and aging on brain functional networks by enhancing “cognitive reserve.” A study by Fan Nils Yang et al. (56) found that individuals with higher educational levels exhibit stronger functional network connectivity in regions such as the dorsal sensorimotor network (dSMN) and the medial temporal lobe (MTL). Education may enhance brain plasticity and compensatory mechanisms, thereby slowing cognitive decline, making it an important potential neurocognitive protective factor for individuals with HIV infection.

#### 4.2.4 The impact of immune status

Immune status plays a crucial role in the organization and remodeling of brain functional networks in individuals with HIV infection. Minosse et al. (57) revealed that PLWH without neurological symptoms exhibit reduced local efficiency (e.g., in the right lingual gyrus and right prefrontal cortex) and deviations in overall network structure (elevated network disruption index), which are closely associated with immune status (CD4^+^ counts). Studies indicate that individuals with lower CD4^+^ counts show greater deviations in brain network structure, highlighting the critical role of the immune system in the remodeling of functional networks.

In summary, aging, substance use, educational attainment, and immune status significantly influence brain functional network connectivity in individuals with HIV infection. Improvements in immune status, particularly the recovery of CD4^+^ counts, as well as higher educational attainment, may play a critical role in mitigating cognitive decline. Meanwhile, the impact of different substances on brain networks exhibits complex variability, warranting further in-depth investigation.

### 4.3 The relationship between functional network abnormalities and cognitive decline

#### 4.3.1 Local efficiency and global network integration

Chockanathan et al. reported that local efficiency changes in people with HIV, particularly in the PCC of the DMN, diminish overall network integration (58). Ventura et al. further observed that functional reorganization in the PCC may serve as a compensatory mechanism, initially helping to maintain attention and working memory. However, as HAND progresses, this compensatory mechanism gradually fails. The decline in PCC efficiency is negatively correlated with deterioration in abstract and executive functioning (59). In addition, using large-scale Granger causality (lsGC) analysis, Chockanathan et al. found that abnormal functional connectivity between the PCC in the DMN and subcortical structures such as the caudate nucleus is closely associated with working memory and executive function impairments in HAND patients (58).

#### 4.3.2 The association between multiple network abnormalities and cognitive performance

In a study by Chaganti et al., rs-fMRI was used to assess functional connectivity changes in patients with HAND. The study included 18 HAND patients in a virologically suppressed state, and the results showed significantly reduced functional connectivity in the SN and the CEN, particularly in the prefrontal cortex and insular regions. These abnormalities were closely associated with impairments in executive function and attention in HAND patients. Moreover, the study found a significant positive correlation between functional connectivity and overall cognitive performance, which was most pronounced in the SN and CEN (51).

Building upon NBS and MCA, Abidin et al. further revealed that the characteristics of disrupted subnetworks between the DMN and other functional networks (e.g., the executive control network) directly affect overall cognitive performance. This disruption is particularly associated with declines in core cognitive domains such as attention, executive function, and processing speed (49).

### 4.4 The diagnostic value of fluctuations in dFC and predictive models

Using a sliding window approach, our team previously quantified changes in dynamic functional connectivity and discovered marked instability in the DMN, CEN, and the FPN. These fluctuations correlate with impairments in cognitive functions such as working memory, attention, and processing speed. Notably, the dynamic instability of DMN functional connectivity can be detected in the early stages of HAND, suggesting the possibility of a potential early diagnostic biomarker (60).

In addition, a study by Yang et al., based on connectome-based predictive modeling (CPM), confirmed its applicability in diagnosing HAND. By using fMRI data to generate whole-brain functional connectivity matrices, the study successfully predicted the global cognitive ability (Global Mean T-score, GMT) of individuals living with HIV. Results showed that the positive network functional connectivity strength in HAND patients was significantly lower than that in non-HAND patients and healthy controls, whereas negative network strength was markedly elevated, reflecting network reorganization and compensatory mechanisms. Key regions in the positive network included the right cerebellum, posterior insular cortex, and putamen, while connections in the negative network involved the right orbitofrontal cortex and the left middle temporal lobe-areas associated with executive function, attentional regulation, and compensatory mechanisms. Longitudinal data analysis revealed a positive correlation between changes in positive network strength and improvements in cognitive function, indicating that functional connectivity characteristics can be used to dynamically monitor cognitive changes in HAND (61). Moreover, network dominance may serve as a potential biomarker to support HAND diagnosis.

### 4.5 Prospects for intervention and treatment

Using a prospective study design, Jia et al. (62) investigated the effects of working memory training (WMT) on the resting-state brain functional networks of PLWH, enrolling 53 PLWH and 53 healthy controls. Results showed that at baseline, the PLWH group had elevated eigenvector centrality in the ventral default mode network (vDMN), indicating network abnormalities. Following WMT intervention, vDMN centrality significantly decreased after 1 month, approached normalization, and remained stable at 6 months. Increases in local efficiency and other network metrics reflected improvements in network segregation and integration. These changes were closely associated with improvements in working memory and general memory capacity, suggesting that WMT may facilitate cognitive recovery by modulating DMN function.

### 4.6 Validation and complementarity of functional connectomics with previous fMRI studies

HIV-induced neuro-synaptic degeneration and aberrant synaptic pruning increase the vulnerability or even disruption of neural circuits, thereby affecting overall neural function and cognitive abilities (63, 64). Traditional rs-fMRI has provided abundant clues for exploring the neuropathological mechanisms of HAND, and this technique is utilized to investigate functional integration and segregation. Functional integration primarily reflects the interactive coordination among different brain regions; in individuals with HIV infection, reductions are mainly observed in frontal-occipital-striatal functional connectivity, and DMN functional connectivity is significantly reduced in HAND patients (24, 53, 65). Functional segregation mainly reflects the intrinsic function of each brain region. Studies have shown that, in the early stages of HIV infection, functional segregation metrics decrease primarily in the frontal lobe but increase in posterior brain regions (e.g., the occipital lobe, precuneus), potentially indicating a compensatory mechanism employed by the brain to adapt to damage (66–69). Through a systematic analysis of whole-brain networks, functional connectomics provides further quantification and validation of these rs-fMRI findings. Research demonstrates that the reduced frontal local efficiency and weakened DMN integration revealed by functional connectomics align closely with the functional integration and segregation abnormalities observed in rs-fMRI. In addition, the enhanced functional segregation in posterior brain regions is considered a compensatory mechanism, and functional connectomics more precisely captures the dynamic changes in this compensatory process. This integrated research framework, which examines regional activity and whole-brain network integrity, enables a more comprehensive understanding of HIV-associated brain network reorganization and provides objective scientific evidence for the early diagnosis and pathological mechanism studies of HAND.

## 5 Core findings of structural connectomics in HAND

### 5.1 Alterations in brain structural network properties and their compensatory mechanisms

#### 5.1.1 Changes in global and nodal network properties

Using DTI data, Liu et al. investigated the differences in brain structural connectivity among PLWH - divided into an ART-treated group and an untreated group - and healthy controls. Their findings include (1): The association between changes in nodal efficiency (Ne) and cognitive function: HIV infection significantly reduced Ne in frontal and temporal regions [e.g., right superior frontal gyrus (SFGmed), olfactory cortex (OLF), and temporal pole (TPOsup)]. Decreased Ne was strongly correlated with declines in attention, working memory, and executive function (2). Compensatory mechanism: In some areas (e.g., SFGmed and OLF), patients receiving ART exhibited increased betweenness centrality (Bc) as a compensatory response, suggesting that ART provides a certain neuroprotective effect on network function in specific regions, though it is insufficient to fully reverse structural abnormalities (3). Multilevel involvement of gray and white matter: Alterations in Ne in the frontal lobe were associated with decreased fractional anisotropy (FA) in corpus callosum white matter tracts, reinforcing the idea that HAND-related neuro-network damage entails multilevel pathologies involving both gray and white matter (70).

Bell et al. (71) examined global network properties in PLWH and their associations with cognitive function. Their findings include: (1) Global network characteristics: PLWH showed a significant reduction in the global clustering coefficient (based on FA), indicating decreased network segregation and reduced local information processing efficiency. Moreover, lower nadir CD4 cell counts correlated with even lower global clustering coefficients, suggesting that more severe historical immunosuppression is linked to reduced network segregation. (2) Nodal characteristics: PLWH demonstrated a significant decrease in nodal degree (based on mean diffusivity, MD) in the left thalamus, suggesting that structural changes in this central relay hub may play a pivotal role in HAND.

#### 5.1.2 Compensatory mechanisms and longitudinal observations

In a “rich club” analysis of the structural network in PLWH patients with complete plasma viral suppression, Aili et al. noted the following: (1) Compensatory enhancement. The local network efficiency (e.g., degree centrality (Dc), Bc, Ne) in PLWH was generally reduced, particularly in the occipital lobe and limbic system regions. However, the Bc and normalized clustering coefficient in regions including the occipital lobe, frontal lobe, insular cortex, and thalamus were elevated, potentially reflecting a compensatory enhancement mechanism (72). (2) Longitudinal study: In patients with ANI, the Ne in the pars triangularis showed a significant decline after a 1.5-year follow-up, yet the small-world properties of the overall brain network remained intact. This outcome may be attributed to neuronal remodeling, functional compensation, and the protective effects of combination antiretroviral therapy (cART), all of which collectively help maintain network stability (73).

### 5.2 The association between brain structural networks and emotion

Caceres and colleagues investigated the relationship between brain structural networks and emotion in youth with perinatally acquired HIV (YPHIV). Using structural MRI data and morphological similarity networks, they examined how brain structural networks relate to emotional symptoms. The results indicated that YPHIV have significantly reduced global efficiency in the whole-brain network, the DMN, and the SN, accompanied by decreased assortativity in the DMN. This reduction in network integration and resilience was significantly associated with emotional symptoms, suggesting that abnormalities in these networks could impact emotion regulation. In addition, inflammatory markers were closely linked to the characteristics of brain structural networks, pointing to a potential role of systemic inflammation in brain network development and emotion regulation. By applying structural connectomics methods, this study provides new insight into how HIV infection influences brain networks and emotional symptoms (74).

### 5.3 Validation and complementarity of structural connectomics with previous sMRI and DTI studies

Structural connectomics, by analyzing the topological features of brain networks and the connectivity among regions, offers a new perspective on understanding the pathological mechanisms of HAND and complements sMRI and DTI findings. (1) Traditional sMRI has revealed subtle gray matter alterations, showing that the virus initially affects subcortical structures such as the putamen, caudate nucleus, and thalamus. As the disease progresses, it further impacts the entorhinal cortex, hippocampus, and association cortices linked to the DMN (66, 75). DTI provides deeper insights into white matter fiber damage, including decreased FA and increased MD in the corpus callosum and corona radiata (76). These changes correlate with immune status and also reflect the degree of neuroinflammation and cognitive impairment (77). Studies employing structural connectomics have found that HIV infection reduces nodal efficiency and global network properties in key regions (such as the thalamus, parietal lobe, frontal lobe, and posterior cingulate cortex), closely aligning with the regional atrophy observed in sMRI and white matter fiber damage revealed by DTI. Meanwhile, the combined effect of decreased network integration and heightened local compensation provides new insight into the functional implications of these structural changes. Although ART confers some protective effect on the stability of brain networks, its efficacy remains to be further optimized (70, 73).

Overall, structural connectomics uncovers both the damage and compensatory mechanisms within the brain networks of HAND patients, highlighting the importance of key regions such as the frontal lobe, thalamus, and DMN in HAND-related cognitive dysfunction. Changes in nodal efficiency and global network properties can serve as potential biomarkers for early HAND diagnosis and disease monitoring. The protective effects of ART can help slow further cognitive decline, though intervention strategies must be optimized to enhance therapeutic outcomes.

## 6 Exploration and discoveries of multimodal imaging combined with connectomics in HAND

Both structural and functional connectomics offer unique perspectives on the imaging characteristics of HAND-related brain damage, yet there may be a mapping relationship between changes in brain function and brain structure. Compared with single-modality research, multimodal imaging can fully integrate these complementary data. It not only enables a more comprehensive analysis of the associations between neurocognitive function and brain structure/function, but also provides novel tools for an in-depth understanding of complex neural network mechanisms (78).

### 6.1 Multimodal connectomics research in the strict sense

Li et al. (79) proposed an improved CPM by integrating multimodal neuroimaging data (structural connectivity [SC] and functional connectivity [FC]) with clinical characteristics. This model successfully predicted the cognitive function (global T-score) of PLWH. The findings were as follows: (1) Complementarity of SC and FC: Combining SC and FC significantly enhanced predictive accuracy and was more sensitive to cognitive changes. (2) Reinforcing effect of clinical data: Incorporating HIV-related clinical features (e.g., viral suppression status, CD4^+^ T-cell counts) further boosted the model’s predictive power. (3). Relationship between functional networks and cognition: Higher cognitive function was associated with stronger connectivity in the subcortical network, cerebellar network, and DMN, whereas lower cognitive function was closely linked to reduced connectivity in the sensorimotor network. This model demonstrated good generalizability in an independent validation cohort and ensured stability through leave-one-out cross-validation (LOOCV). Although the sensitivity of SC and heterogeneity in validation data limited certain aspects of performance, future improvements through high-resolution imaging and longitudinal data may further optimize the model, providing a potential tool for early HAND diagnosis and precision intervention.

### 6.2 Additional studies incorporating multimodal imaging

Beyond the strict sense of multimodal connectomics research, numerous studies-despite not constructing a full-brain multimodal connectome-have integrated structural and functional imaging data in ways that closely align with the principles of multimodal connectomics. This approach offers valuable insights into the neural mechanisms underlying HAND.

#### 6.2.1 Exploring the combination of gray matter volume and functional connectivity

Liu and colleagues used structural MRI and functional MRI to investigate the dual impact of HIV infection on gray matter volume and resting-state functional connectivity. The results showed significant reductions in gray matter volume in the thalamus, occipital lobe, and hippocampal/parahippocampal regions among individuals with HIV infection. Meanwhile, functional connectivity between the visual cortex and the DMN decreased, and functional connectivity in the thalamic-prefrontal pathway and between the thalamus and somatosensory association cortex also underwent marked changes. Individuals with cognitive impairment (the HAND group) showed a significant reduction in functional connectivity between the left thalamus and the right dorsolateral prefrontal cortex. The study indicates that brain atrophy and functional reorganization caused by HIV infection are closely linked to declines in neurocognitive function (66).

#### 6.2.2 Structural connectivity damage and functional connectivity compensation

Shana A Hall and colleagues found that people living with HIV show significantly reduced structural integrity in the mid-posterior corpus callosum (indicating structural connectivity damage). However, fMRI data revealed increased functional connectivity in certain networks (such as the DMN and the prefrontal network), which may represent a compensatory mechanism for structural damage. The study suggests that injury to the corpus callosum could trigger functional compensation, helping to maintain cognitive function (80).

#### 6.2.3 Microstructural damage and functional connectivity compensation

Previous research, using a whole-brain computational model (the reduced Microstructure-informed Functional Model, rMFM), investigated the dual impact of HIV infection on brain microstructure and functional connectivity. By integrating diffusion-weighted MRI (DWI) and fMRI data, microstructural information (e.g., axonal density) was combined with functional connectivity data to capture the complex relationship between structural disruption and network reorganization. The results indicated that individuals with HIV infection exhibit significant microstructural damage in regions such as the frontal and temporal lobes, accompanied by a reorganization of functional connectivity. In certain networks (such as the DMN and the task-positive network), functional connectivity was enhanced, while it weakened in other networks, possibly indicating a compensatory mechanism. Microstructural damage was closely associated with declines in cognitive function, particularly in attention, memory, and executive domains. The study also evaluated the effects of cART, finding that 12 weeks of treatment partially alleviated abnormalities in both microstructure and functional connectivity, accompanied by partial recovery of cognitive function. By integrating microstructural information and functional network data via rMFM, this research is among the first to illuminate the complex interplay between microstructure and functional networks, offering new insights for personalized treatment and early intervention (81).

In summary, multimodal imaging studies that integrate SC and FC have revealed both damage and compensatory mechanisms in the brain structure and functional networks of HAND patients. Common findings include: (1) Functional–structural complementarity: functional connectivity is more sensitive to cognitive changes, while structural connectivity more reliably reflects long-term damage; (2) Compensatory mechanisms: the enhancement of certain functional networks may serve as compensation for structural damage; and (3) Clinical application potential: combining multimodal imaging with clinical data could be a valuable tool for early HAND diagnosis and precise intervention.

## 7 Summary of current research status and future directions

This review analyzes the brain network characteristics of HAND from a connectomics perspective. Most studies administered cognitive assessments to participants and attempted to correlate the results with imaging data. Notably, whether or not researchers have distinguished among HAND subtypes, they have consistently highlighted the critical role of marked structural and functional brain network abnormalities in diagnosing cognitive impairment in HIV-infected individuals. However, there remains a lack of research on the specific brain network changes in different HAND subtypes (e.g., ANI, MND, HAD), particularly in mid-to-late stages such as MND and HAD. Current studies mainly focus on early subtypes like ANI, which, although revealing some early-stage HAND network features, provide limited connectomics-based insights into later subtypes-an important limitation that hinders a comprehensive understanding of the pathological progression of HAND.

**Figure 3:**
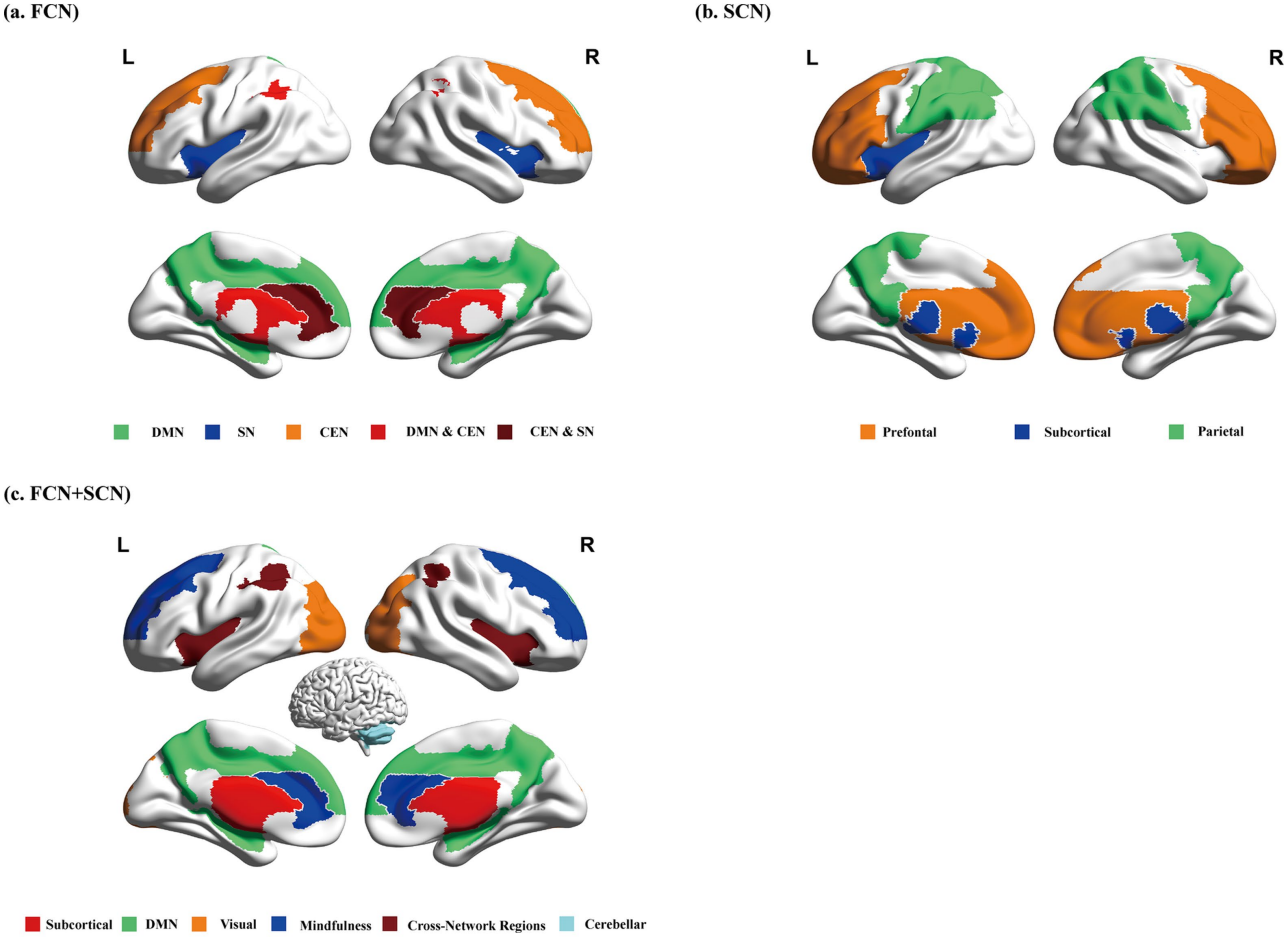
Unimodal and multimodal connectomics reveal disease progression patterns from different dimensions. **(A)** Functional connectomics: Abnormal topological properties are primarily concentrated in the DMN, SN, and CEN. Some brain regions may simultaneously participate in multiple network functions, particularly the prefrontal and parietal cortices. These cross-network regions are marked in dark red and red in the figure; **(B)** Structural connectomics: Abnormal topological properties are mainly distributed in the prefrontal network, parietal network, and subcortical networks, highlighting critical abnormalities in structural connectivity; **(C)** Multimodal connectomics: Although the results of strict multimodal connectomics studies may differ from those aligned with the connectomics framework, studies widely highlight abnormalities in the subcortical network, DMN, occipital cortex-dominated visual network, task-positive network, and cerebellar network. These findings underscore their significant roles in disease progression. Cross-network regions are also marked in dark red. FCN, functional network connectivity; SCN, structural network connectivity; DMN, default mode network; SN, salience network; CEN, central executive network.

The diagnosis of HAND relies on cognitive assessments, and existing research has already demonstrated the pivotal role of structural and functional brain network abnormalities in diagnosing HAND (as illustrated in Figure 3). However, varying sample characteristics (e.g., treatment status, disease course, degree of immunosuppression), imaging analysis methods, and sample sizes produce substantial heterogeneity across studies, limiting the generalizability of their findings. Nevertheless, these studies underscore several key discoveries: patients with HAND at different stages exhibit distinct brain network features, and the pattern of damage shows emerging regularities that complement traditional imaging findings. While ART positively contributes to maintaining brain network stability and slowing cognitive decline, it has yet to fully reverse the observed abnormalities.

Connectomics research in HAND remains in an exploratory phase with relatively few published studies, yet its potential is becoming increasingly evident. Future investigations should fully leverage the strengths of multimodal connectomics, integrating cognitive assessments and dynamic neuroimaging data to further clarify the connectomics-based features of specific HAND subtypes. By uniting large-scale, multicenter international collaborations with advanced techniques such as dynamic network analysis and machine learning, and by conducting longitudinal studies, it will be possible to uncover how treatment-related compensatory mechanisms evolve along with brain network properties. Such efforts not only deepen our understanding of the multifaceted impact of HIV infection on brain function and microstructure but also provide vital guidance for precise diagnosis and individualized treatment of HAND, offering new hope for preserving cognitive health and improving the quality of life in HIV-infected individuals.
